# Robotic-Assisted Hip and Knee Arthroplasty: A Bibliometric Analysis Using the Scopus Database

**DOI:** 10.7759/cureus.56617

**Published:** 2024-03-21

**Authors:** Ren Yi Kow, Rizal Abdul Rani, Mohamad Hilmi Mohamad Nazarallah, Juzaily F Leong, Muhammad Fathi Hayyun, Chooi Leng Low, Ahmad Hafiz Zulkifly, Nor Hamdan Mohamad Yahaya

**Affiliations:** 1 Department of Orthopedics, Traumatology, and Rehabilitation, International Islamic University Malaysia, Kuantan, MYS; 2 Department of Orthopedics and Traumatology, Universiti Kebangsaan Malaysia, Kuala Lumpur, MYS; 3 Department of Orthopedics, Universiti Sains Islam Malaysia, Nilai, MYS; 4 Department of Orthopedics and Traumatology, Hospital Canselor Tuanku Muhriz, Kuala Lumpur, MYS; 5 Department of Radiology, International Islamic University Malaysia, Kuantan, MYS

**Keywords:** bibliometric analysis, hip arthritis, knee arthritis, robotic hip and knee surgery, robotic knee surgery

## Abstract

Robotic-assisted hip and knee arthroplasty represents cutting-edge advancements in orthopedic surgery, harnessing robotic technology to enhance precision, improve clinical outcomes, and facilitate intra-operative procedures. In these robotic-assisted surgeries, the robotic systems assist surgeons in planning and executing joint replacement surgeries, thereby facilitating personalized implant positioning and optimizing the fit and alignment of hip and knee implants. Despite the increasing attention garnered by robotic-assisted hip and knee arthroplasty in recent years, a comprehensive bibliometric analysis using the Scopus database has yet to be conducted. This bibliometric analysis reviews the Scopus database from 1961 until 2022 to investigate the literature on the field of robotic-assisted hip and knee arthroplasty. A total of 577 articles that satisfied the selection criteria were included in this review. The majority of the articles focus more on total knee replacement, compared to total hip replacement and unicompartmental knee arthroplasty. The overwhelming majority of the articles were authored by researchers and clinicians from the United States of America (USA) and the United Kingdom (UK). Similarly, most of the articles with the highest number of citations were authored by researchers and clinicians from these regions. This comprehensive bibliometric analysis using Scopus in the domain of robotic-assisted hip and knee replacement has the potential to act as a roadmap for researchers, clinicians, and policymakers, facilitating informed decision-making, promoting collaborative initiatives, and guiding the development of future studies to further advance the field of robotic-assisted hip and knee arthroplasty.

## Introduction and background

With the advancement of technology and healthcare systems, the proportion of the world’s population over 60 years old is estimated to be 22% [1-3]. As the population ages, the incidence of degenerative joint diseases also increases accordingly [4-7]. As a result, the demand for restorative surgeries, such as knee and hip replacements, is projected to rise in tandem with the increasing prevalence of patients with hip and knee osteoarthritis [7-9]. In 2010, an estimated 2.5 million people underwent total hip replacement, while 4.7 million people underwent total knee replacement (TKR) in the United States of America (USA) [7,8]. According to projections by Kurtz et al., the demand for primary total knee arthroplasties is expected to grow by more than 670% to reach 3.48 million procedures by 2030 [9]. A similar upward trend is anticipated for the demand for primary total hip arthroplasties, projected to increase by 174% to 572,000 procedures by 2030 [9].

Robotic arthroplasty represents a groundbreaking technological leap forward in the realm of orthopedic surgery, potentially transforming the approach to joint replacement surgeries [10,11]. This innovative method utilizes preoperative imaging, such as computed tomography scans, to generate three-dimensional models of a patient’s anatomy, providing surgeons with unparalleled precision and planning capabilities [11,12]. Robotic systems play a pivotal role in optimizing the accuracy of implant placement and ensuring the consistency of clinical outcomes [10-12]. Early results of robotic-assisted joint replacement surgeries demonstrate potential benefits, including improved postoperative comfort, reproducibility, and a reduction in outliers [10-12]. Despite a few reports of complications such as pinhole fractures, infections, and iatrogenic injuries, the increasing acceptance of robotic-assisted joint replacement underscores the promising trajectory of this technology in revolutionizing joint arthroplasty and shaping the future landscape of orthopedic surgery [11].

Bibliometric analysis is an effective method for evaluating the scholarly impact of published literature [13-15]. It serves to summarize research interests, measure relationships and clustering within a research field, and assess academic quality and impact by measuring the number of publications and citations each article received [15-19]. Additionally, it can reveal any collaboration among authors, institutions, and countries, which can be instrumental in forming new collaborative projects with researchers of similar expertise. Furthermore, bibliometric analysis can identify research gaps within a research field, providing valuable insights and guidance to researchers and institutions. To date, bibliometric analysis of robotic-assisted hip and knee arthroplasty has been performed [10,11,13-16]. Nevertheless, these analyses solely rely on the Web of Science Core Collection database for review [10,11,13-16,19]. Our review aims to conduct a comprehensive bibliometric analysis of the literature using the Scopus database on robotic-assisted hip and knee arthroplasty.

## Review

Literature search and search strategy

The literature on robotic-assisted hip and knee arthroplasty was sourced from the Scopus database. The search strategy was formulated using the following keywords: (robot* OR rob*) AND (replace* OR arthr*). Keywords such as “hip” and “knee” were deliberately omitted from the search term to prevent the exclusion of articles with non-specific keywords. The search spanned from January 1, 1961, to December 31, 2022. The retrieved articles underwent screening by the two principal authors for suitability. Only English-language articles related to robotic-assisted hip and knee arthroplasty were included. Other types of publications, such as conference proceedings, book chapters, trade journals, and errata, were excluded. Arthroplasty of other joints such as the shoulder, elbow, hand, and ankle was also excluded.

The retrieved data were processed using R Studio 2021 for Windows (The R Foundation), with the “bibliometrix” package (K-Synth Srl, Naples, Italy) installed in R [17]. Information such as authorship, title, publication, number of citations, affiliations, journal source, references, and keywords were extracted using the R software. Data presentation, including illustrative graphs, was completed using “bibliometrix,” while Microsoft Excel 2019 (Microsoft Corporation, Redmond, WA, USA) was employed for data organization.

Results

Annual Scientific Production and Journal

**Figure 1:**
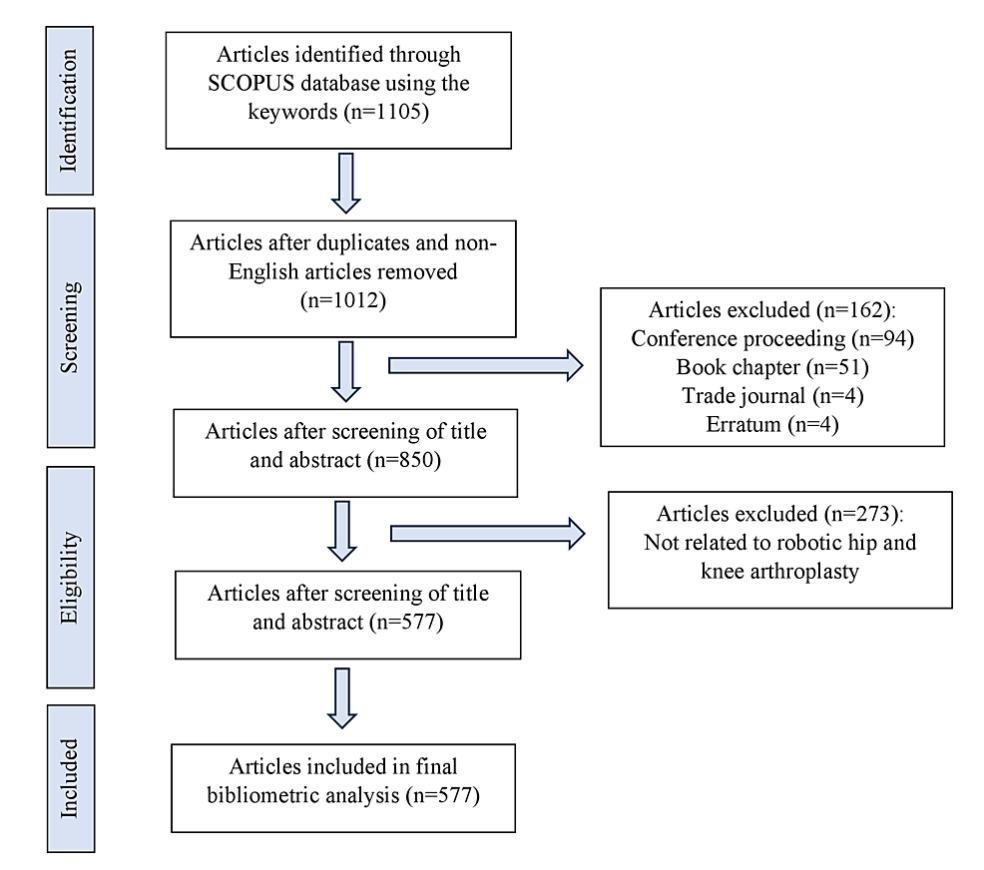
Flowchart of search results

**Figure 2:**
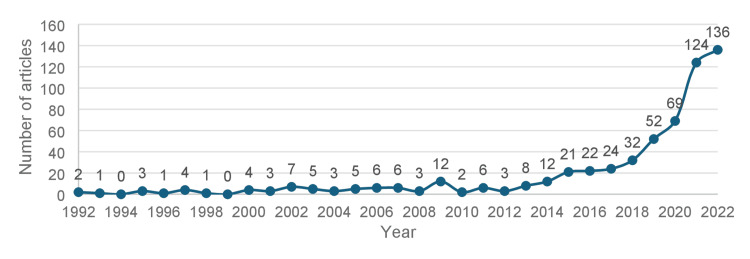
Number of articles published in each year Note: Graph converted from bibliometrix software in R studio and presented using Microsoft Excel

A total of 1105 articles were retrieved from the Scopus database from 1961 to 2022, using the terms presented (Figure 1). After screening by the lead authors, only 577 articles met the inclusion criteria and were analyzed in this review. As depicted in Figure 2, the first related article was published in 1992, and the number of annual publications remained low until 2014, when it began to slowly increase. From 2018 onwards, the field experienced exponential growth in annual publications, with a total of 136 related articles published in 2022.

A total of 577 articles were published in various journals within the study period. Table 1 presents the top 10 journals with the highest number of publications in the field of robotic-assisted hip and knee arthroplasty. The Journal of Arthroplasty leads the table with 50 publications. This was followed by the Journal of Knee Surgery (40 publications), International Journal of Medical Robotics and Computer Assisted Surgery (27 publications), Surgical Technology International (27 publications), Arthroplasty Today (22 publications), Bone and Joint Journal (22 publications), Knee Surgery, Sports Traumatology, Arthroscopy (22 publications), Chinese Journal of Reparative and Reconstructive Surgery (17 publications), American Journal of Orthopedics (13 publications), and Achieves of Orthopaedic and Trauma Surgery (13 publications). Notably, among the top 10 journals, two (the International Journal of Medical Robotics and Computer-Assisted Surgery and Surgical Technology International) are dedicated to technology and robotic surgery.

**Table 1:** Top 10 journals with the most number of articles

Journal name	Number of articles	Percentage
Journal of Arthroplasty	50	8.7
Journal of Knee Surgery	40	6.9
International Journal of Medical Robotics and Computer-Assisted Surgery	27	4.7
Surgical Technology International	27	4.7
Arthroplasty Today	22	3.8
Bone and Joint Journal	22	3.8
Knee Surgery, Sports Traumatology, Arthroscopy	22	3.8
Chinese Journal of Reparative and Reconstructive Surgery	17	2.9
American Journal of Orthopedics	13	2.3
Achieves of Orthopaedic and Trauma Surgery	13	2.3

Authors and Their Country of Origin

Table 2 summarizes the top 10 authors with the highest number of publications in the field of robotic-assisted hip and knee arthroplasty. Mont MA had the highest number of publications, with a total of 47 articles. This was followed by Haddad FS (23 publications), Sodhi N (21 publications), Kayani B (19 publications), Lonner JH (19 publications), Khlopas A (18 publications), Pearle AD (16 publications), Chai W (14 publications), Domb BG (14 publications), and Konan S (14 publications).

**Table 2:** Top 10 authors with the most number of publications

Author’s name	Number of articles	Percentage
Mont MA	47	8.1
Haddad FS	23	4.0
Sodhi N	21	3.6
Kayani B	19	3.3
Lonner JH	19	3.3
Khlopas A	18	3.1
Pearle AD	16	2.8
Chai W	14	2.4
Domb BG	14	2.4
Konan S	14	2.4

Table 3 demonstrates the top 10 countries with the highest number of publications in the field of robotic-assisted hip and knee arthroplasty. The USA overwhelmingly leads in the number of publications in this field, with a total of 162 articles attributed to authors from the USA. This was followed by the UK (61 publications), China (43 publications), Germany (22 publications), Australia (19 publications), Italy (17 publications), France (14 publications), Korea (14 publications), Japan (12 publications), and India (10 publications). Among the top 10 countries, only two of them had a multiple country production (MCP) ratio of more than 0.2 (20%). Up to 71% of the articles published by authors from France had collaborated with another country.

**Table 3:** Author’s country with the most number of publications SCP: single country production, MCP: multiple country production

Country	Number of articles	Percentage	SCP	MCP	MCP ratio
USA	162	28.1	145	17	0.105
UK	61	10.6	55	6	0.098
China	43	7.5	35	8	0.186
Germany	22	3.8	20	2	0.091
Australia	19	3.3	15	4	0.211
Italy	17	2.9	14	3	0.176
France	14	2.4	4	10	0.714
Korea	14	2.4	13	1	0.071
Japan	12	2.1	11	1	0.083
India	10	1.7	9	1	0.100

Type of Articles

With regard to the types of articles, over 80% involved clinical research. Approximately 40% of research publications were attributed to clinical research on TKR. This was followed by clinical research involving total hip replacement (23.1%) and unicondylar (unicompartmental) knee arthroplasty (UKA) (18.4%). A similar trend was observed in review and non-clinical articles, where TKR articles were predominant (3.1% and 1.9%, respectively). In contrast to clinical research, the number of reviews and non-clinical articles involving UKA was higher than THR (2.6% vs. 1.9% and 1.4% vs. 1.0%, respectively). Table 4 summarizes the types of articles reviewed in this bibliometric analysis. About 6% of the articles were not categorized into any of these groups, with the majority of them reviewing the use of robotic-assisted surgery in both TKR and THR or all TKR, THR, and UKA.

**Table 4:** Categorization of the articles THR: total hip replacement, TKR: total knee replacement, UKA: unicondylar (unicompartmental) knee arthroplasty

Type of articles	Number of articles	Percentage
Non-clinical (THR)	6	1.0
Non-clinical (TKR)	11	1.9
Non-clinical (UKA)	8	1.4
THR (clinical)	133	23.1
TKR (clinical)	234	40.6
UKA (clinical)	106	18.4
Review (THR)	11	1.9
Review (TKR)	18	3.1
Review (UKA)	15	2.6
Others	35	6.0
Total	577	100

Top 10 Articles With the Most Citations

Table 5 shows the top 10 articles with the most citations within the study period. The article with the title “Primary and revision total hip replacement using robodoc system” by Bargar WL from the USA had the highest number of citations (317 citations) [20]. “Hands-on robotic unicompartmental knee replacement: a prospective, randomised controlled study of the acrobot system” by Cobb J from the UK had the second-highest number of citations (266 citations) [21]. This was followed by “Development of a surgical robot for cementless total hip arthroplasty” by Paul HA (257 citations), “Robotics in arthroplasty: a comprehensive review” by Jcofsky DJ (244 citations), “Improved accuracy of component positioning with robotic-assisted unicompartmental knee arthroplasty: data from a prospective, randomized controlled study” by Bell SW (193 citations), “Technique and first clinical results of robot-assisted total knee replacement” by Siebert W (190 citations), “Comparison of robotic-assisted and manual implantation of a primary total hip replacement. A prospective study” by Honl M (182 citations), “Robotic-arm assisted total knee arthroplasty is associated with improved early functional recovery and reduced time to hospital discharge compared with conventional jig-based total knee arthroplasty: a prospective cohort study” by Kayani B (180 citations), “Simultaneous bilateral total knee arthroplasty with robotic and conventional techniques: a prospective, randomized study” by Song EK (157 citations), and “Robotic-arm assisted total knee arthroplasty has a learning curve of seven cases for integration into the surgical workflow but no learning curve effect for accuracy of implant positioning” by Kayani B (154 citations) [22-29].

**Table 5:** Top 10 articles with the most citations THR: total hip replacement, UKA: unicondylar (unicompartmental) knee arthroplasty, TKR: total knee replacement

Number	Citation	First author	Country	Title	Year	Journal	Type
1.	317	Bargar WL [20]	USA	Primary and revision total hip replacement using Robodoc system	1998	Clinical Orthopaedics And Related Research	THR
2	266	Cobb J [21]	UK	Hands-on robotic unicompartmental knee replacement: a prospective, randomised controlled study of the acrobot system	2006	Journal of Bone & Joint Surgery British Volume	UKA
3	257	Paul HA [22]	USA	Development of a surgical robot for cementless total hip arthroplasty	1992	Clinical Orthopaedics And Related Research	THR
4	244	Jacofsky DJ [23]	USA	Robotics in arthroplasty: a comprehensive review	2016	Journal of Arthroplasty	Review
5	193	Bell SW [24]	UK	Improved accuracy of component positioning with robotic-assisted unicompartmental knee arthroplasty: data from a prospective, randomized controlled study	2016	Journal of Bone & Joint Surgery American Volume	UKA
6	190	Siebert W [25]	Germany	Technique and first clinical results of robot-assisted total knee replacement	2002	Knee	TKR
7	182	Honl M [26]	Germany	Comparison of robotic-assisted and manual implantation of a primary total hip replacement. A prospective study	2003	Journal of Bone & Joint Surgery American Volume	THR
8	180	Kayani B [27]	UK	Robotic-arm assisted total knee arthroplasty is associated with improved early functional recovery and reduced time to hospital discharge compared with conventional jig-based total knee arthroplasty: a prospective cohort study	2018	The Bone & Joint Journal	TKR
9	157	Song EK [28]	Korea	Simultaneous bilateral total knee arthroplasty with robotic and conventional techniques: a prospective, randomized study	2011	Knee Surgery, Sports Traumatology, Arthroscopy	TKR
10	154	Kayani B [29]	UK	Robotic-arm assisted total knee arthroplasty has a learning curve of seven cases for integration into the surgical workflow but no learning curve effect for accuracy of implant positioning	2019	Knee Surgery, Sports Traumatology, Arthroscopy	TKR

Collaboration Between Different Countries

Authors from the USA engage in collaborative research and partnerships with researchers from various other countries. They collaborate with researchers from countries such as the UK (eight publications), China (six publications), Israel (five publications), and France (four publications), with two publications from Singapore, India, Australia, and Argentina. This indicates a global and interconnected approach to their work, emphasizing the importance of international collaboration in the advancement of research and the exchange of knowledge.

**Figure 3:**
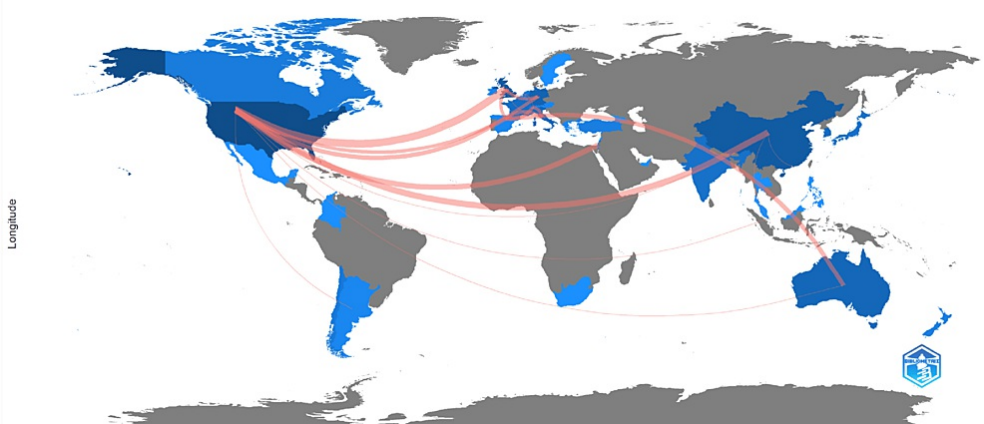
Countries' collaboration world map The pink lines depict the collaboration between countries with the thickness of the pink line indicating the degree of collaboration Note: Figure generated using the bibliometrix software in R studio [17]

Discussion

Published by Elsevier, the Scopus database is a comprehensive repository covering a variety of journals, conference proceedings, and patents [18,19]. In contrast to the PubMed database published by the National Center for Biotechnology Information, which primarily focuses on biomedical and life sciences, the Scopus database is multidisciplinary. It indexes documents from the sciences, social sciences, arts, and humanities. Beyond providing basic information such as authors’ information and abstracts, Scopus also offers comprehensive citation analysis, including h-index and citation counts [18]. As the academic landscape continues to evolve, understanding the nuances of each database becomes crucial, given that each has its strengths and limitations. With this in mind, this bibliometric analysis investigates robotic-assisted hip and knee arthroplasty within the realm of the Scopus database. This review validates a wide range of pertinent information regarding the authors, topics, and time periods that have had a profound impact on the field of robotic-assisted hip and knee arthroplasty.

In this review, we identify 10 articles with the most citations in the Scopus database related to robotic-assisted hip and knee arthroplasty. Citation analysis, albeit with its limitations, allows for an objective measurement of peer recognition and offers insights into the readership of the articles [19,30-32]. It is crucial for researchers to acknowledge that citation rates alone may not fully capture the multifaceted nature of research impact. While not directly linked to study quality, articles with high citation numbers indicate that various researchers have found the content beneficial and its material worthy of inclusion and discussion in their work [30-32]. Only two of the top 10 articles with the most citations were published within the last five years, with the top three articles having been published more than 15 years ago. This is expected, as older articles have had more time to accumulate citations compared to recently published ones. Some researchers prefer to utilize mean citations or citation density (the number of citations over a number of years) to delineate the significance of each article, but we did not delve into this analysis in this review.

Researchers from the USA and UK exhibit a significant trend of publishing more articles and receiving higher citations. Notably, authors from these countries command an overwhelming share in this field in terms of the absolute number of articles published, underscoring their substantial contribution to the global research landscape. Furthermore, seven of the top 10 articles with the most citations were contributed by authors from either the USA or the UK, highlighting the significant impact of research from these countries. Our findings are consistent with the findings of Wan et al., who conducted a bibliometric analysis using the Web of Science Core Collection database [11]. Firstly, the intrinsic nature of the selection bias plays a role, as these countries utilize English as the primary language of scientific communications. In the same vein, articles in the English language tend to have access to a wider global audience, contributing to higher citation rates. The use of English as a medium of communication also fosters international collaborations and networking initiatives with researchers from other parts of the world, facilitating the exchange of ideas and diverse perspectives. Additionally, adequate funding opportunities and access to advanced medical technology provided by well-established institutions in the USA and the UK empower researchers from this region to conduct extensive studies, fostering an environment conducive to impactful research. This is especially evident when eight out of the top 10 sponsoring institutions or companies are based in either the USA or the UK [11].

Limitation

There are several intrinsic limitations to this bibliometric analysis. Firstly, selection bias is present in this review, as only English articles derived from the Scopus database are included. Secondly, owing to the time frame of the literature search, the number of citations received by the articles reviewed in this bibliometric analysis may be potentially higher, especially for articles published in recent years. The wide duration of publication not only affects the number of citations but also introduces variation in the authors’ affiliation, as clinicians and researchers may have moved to another institution or country. Additionally, since only the Scopus database is examined in this bibliometric analysis, the citation count may differ if other databases, such as Web of Science Core Collection or Google Scholar, are used.

## Conclusions

Robotic-assisted hip and knee arthroplasty has been expanding exponentially in the past five years, underscoring the importance of robotic technology in refining precision and improving clinical outcomes. This review offers a detailed account and bibliometric analysis of publications on robotic-assisted hip and knee arthroplasty in the Scopus database, covering a diverse range of durations. These findings provide insights into the current status and research trends, offering valuable guidance for future practices and directions. Despite the increasing number of peer-reviewed articles in this field, certain parts of the world continue to dominate the technology.
